# The 20-year latency: transformation of a remote Müllerian tumour to low-grade serous carcinoma within an incisional hernia

**DOI:** 10.1093/jscr/rjag704

**Published:** 2026-08-12

**Authors:** Maeve Tremis, Jillian Cassidy, John Lucci, Alexandra Shapiro, Seth Kipnis

**Affiliations:** St. George's University School of Medicine, University Centre, True Blue, St. George's, Grenada, West Indies; Department of Surgery, Jersey Shore University Medical Center, Hackensack Meridian Health, Neptune, NJ 07753, United States; Department of Pathology, Jersey Shore University Medical Center, Hackensack Meridian Health, Neptune, NJ 07753, United States; Department of Surgery, Jersey Shore University Medical Center, Hackensack Meridian Health, Neptune, NJ 07753, United States; Department of Surgery, Jersey Shore University Medical Center, Hackensack Meridian Health, Neptune, NJ 07753, United States

**Keywords:** low-grade serous carcinoma, ovarian cancer, late recurrence, incisional hernia

## Abstract

Low-grade serous carcinoma (LGSC) is known for its indolent course and potential for late recurrence, often decades after initial surgery. We present the case of a 62-year-old female who was incidentally found to have LGSC consistent with a delayed Müllerian neoplastic recurrence during an incisional hernia repair, 20 years after her primary gynecologic surgery. This case highlights the phenomenon of extremely delayed recurrence and underscores the need for long-term surveillance and a high index of suspicion for malignancy in patients with a remote history of gynecologic tumors, even when they present with common surgical problems.

## Introduction

Low-grade serous carcinoma (LGSC) of Müllerian origin is marked by a slow clinical course and low-grade cytologic atypia. It has a known tendency toward late, sometimes isolated recurrences along the peritoneal and abdominal wall surfaces [1, 2]. Recurrences can arise more than a decade after the index operation—and, in rare reports, several decades later. This pattern emphasizes the consideration for longer, structured surveillance, rather than standard intermediate-term oncologic surveillance timelines [2–4].

Abdominal wall and port-site metastases have been reported both in advanced intra-abdominal disease and after recent operative manipulation, as well as isolated implants arising long after hysterectomy or oophorectomy for Müllerian neoplasia—including cases where serous borderline tumours (SBT) recur as LGSC [3–6]. The primary pathophysiologic mechanism is thought to be iatrogenic implantation of exfoliated tumour cells into laparotomy or laparoscopy wounds through direct contact, contaminated instruments, ascitic fluid or the ‘chimney effect’ of pneumoperitoneum, followed by prolonged dormancy and very slow local growth, characteristic of the low-grade serous pathway [1, 2, 7, 8]. Several case reports describe anterior abdominal wall metastases manifesting 10–42 years after primary ovarian cancer surgery, supporting the possibility of extremely delayed wound or scar recurrences, and underscoring the importance of ongoing vigilance among surgeons confronted with unusual abdominal wall masses in patients with remote gynecologic cancer histories [3–6].

Within this context, our patient developed a low-grade serous carcinoma involving tissue adjacent to a previous lower midline laparotomy for a gynecologic tumour in 2002. She had not received intervening gynecologic oncology follow-up or adjuvant therapy. This is most consistent with an extremely delayed abdominal wall recurrence from occult Müllerian neoplastic cells implanted during her original open total abdominal hysterectomy [1–6].

## Case presentation

This is a case of a 62-year-old female patient with hereditary hemolytic uremic syndrome, hypertension, obesity, relapsing polychondritis, and rheumatoid arthritis who presented with abdominal pain and vomiting related to an incisional hernia. The patient’s past surgical history was notable for an open total abdominal hysterectomy in 2002 for a gynecologic tumour invading the bladder; she received no adjuvant therapy or subsequent gynecologic follow-up.

The patient had dull, intermittent abdominal discomfort at her hernia site when she was first diagnosed with the hernia 5 years earlier. She presented to the emergency department, where computed tomography (CT) imaging showed a large, incarcerated anterior abdominal wall hernia containing colon and small bowel. This caused a partial obstruction. After medical management and discharge, she was seen in our outpatient clinic. Given her history and persistent symptoms, we recommended surgical repair of the incisional hernia with panniculectomy. The patient underwent the planned procedure, during which a 20 cm hernia defect was identified. Abdominal wall reconstruction was performed with bilateral component separation and a 25×20 cm Phasix mesh was placed. Intraoperatively, the fascial defect was noted to be large, measuring 17  by 8 cm. The subcutaneous tissue and hernia sac appeared grossly unremarkable, and no suspicious macroscopic architectural changes or tumour nodules were observed during the repair. Her postoperative course was complicated by multiple readmissions for wound debridement and wound vac placement but eventually recovered.

## Pathology

**Figure 1:**
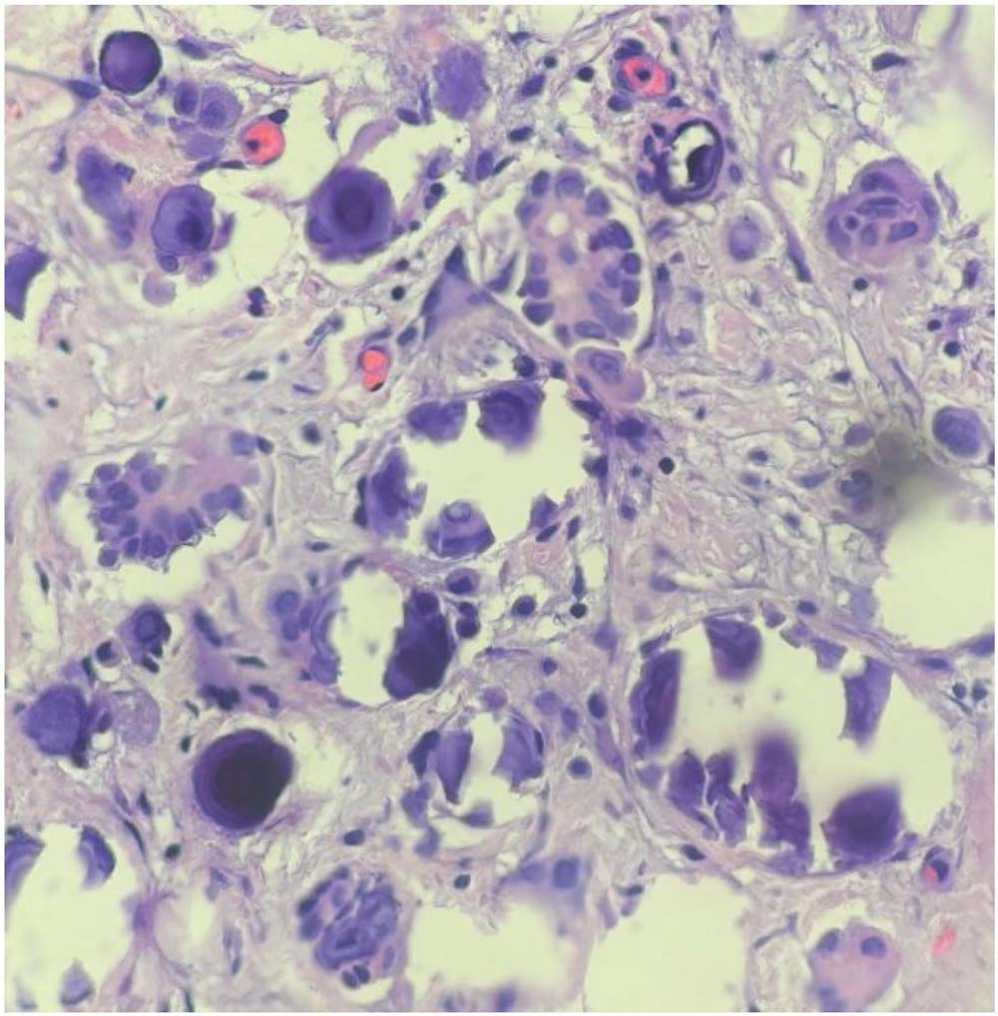
Papillary glandular structures with minimal nuclear pleomorphism are appreciated on high power.

**Figure 2:**
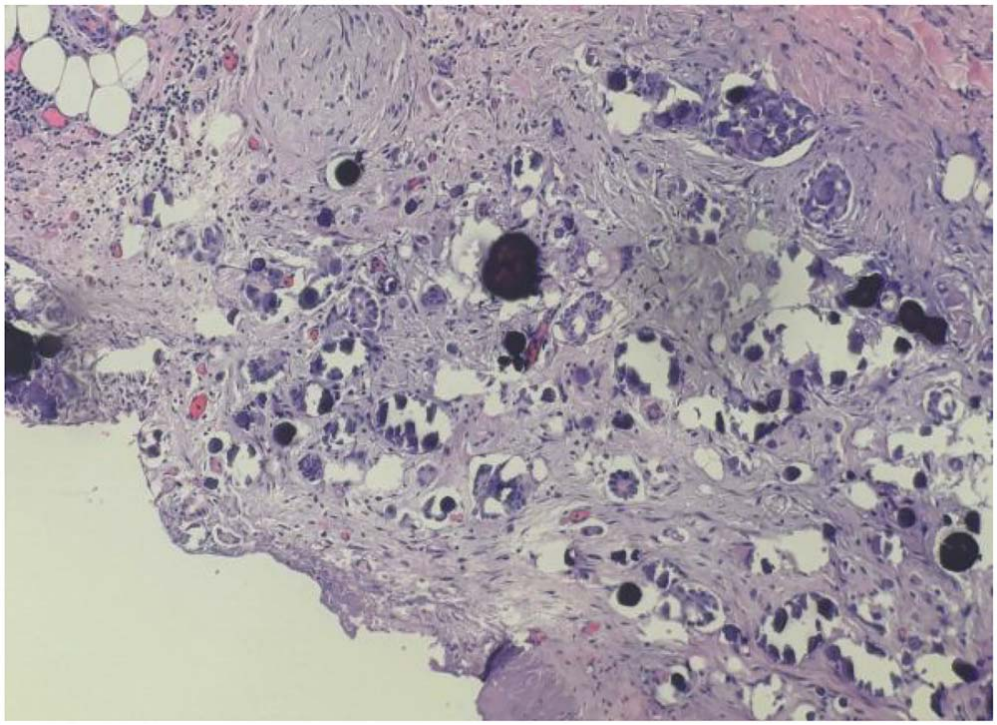
Psammomatous calcifications are observed as acellular concentric laminations.

**Figure 3:**
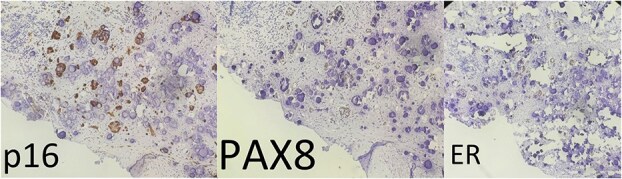
PAX8 & p16 nuclear stain is positive, which is standard. ER stain is positive, which is variable.

Surgical specimen, abdominal wall tissue from the panniculectomy, consisting of skin and subcutaneous tissue and measuring up to 33 × 18 × 9 cm, was sent for pathologic review. Microscopic review of the tissue showed fibroadipose tissue, small papillary structures (Fig. 1), and extensive psammomatous calcifications (Fig. 2) without clear evidence of stromal invasion and low mitotic activity (<12 mitoses per 10 high-power fields). A Ki-67 index was not performed. Immunohistochemistry was positive for AE1/AE3, PAX8, CK7, p16, and ER, and negative for CK20 (Fig. 3). P53 showed wild-type nuclear expression. HER2 by FISH was negative. The specimen surgical resection margins were clear of carcinoma. An expert pathologist confirmed that these findings indicated LGSC. Subsequent tissue from wound debridement showed no carcinoma.

## Postoperative oncologic work-up

Following the definitive histopathologic diagnosis of LGSC, the patient was promptly referred to medical oncology. A comprehensive postoperative staging work-up was initiated, including a baseline serum CA-125 level, which was normal. Comprehensive surveillance cross-sectional imaging with positron emission tomography–computed tomography (PET-CT)/CT of the chest, abdomen, and pelvis demonstrated no evidence of fluorodeoxyglucose avidity, residual localized disease, or intra-abdominal tumour burden. At her most recent follow-up visit, 12 months postoperatively, she remains clinically stable, with no evidence of local or systemic recurrence.

## Discussion

This case highlights an unexpected pathological finding during a common general surgery procedure, 20 years after the patient’s initial hysterectomy. Prior records were unavailable. We hypothesize that the initial diagnosis was aSBT, which makes up 15%–20% of non-benign ovarian neoplasms. SBT can transform into LGSC [9, 10]. Because SBT and LGSC share similar molecular pathways, these tumors may remain dormant and transform slowly over time. This underscores the need to keep them in the differential diagnosis for patients with a remote history of Mullerian pathology [9–12].

The current National Comprehensive Cancer Network guidelines (version 4.2026) for serous borderline tumors recommend surgical resection. If surgery is complete and there is no LGSC, patients are generally observed [13]. Those with LGSC consider adjuvant options based on stage. Observation usually means visits every 3–12 months for 5 years, then as needed [8, 11, 12, 14, 15]. However, the indolent nature and late recurrences raise the question of whether longer or lifelong observation should be considered for higher-risk patients. Diagnostic challenges remain. In this patient, there were no imaging or lab signs that required earlier intervention. The lesion appeared only during hernia repair, demonstrating the diagnostic limitations of macroscopic imaging modalities for microscopic or dormant cellular implants. Notably, her postoperative course was more complicated than is typical for an isolated incarcerated hernia. She needed multiple readmissions, wound debridement, vacuum-assisted closure, and delayed primary closure of a large wound. While delayed wound healing is common in the context of her significant pre-existing comorbidities—including severe obesity, rheumatoid arthritis, and relapsing polychondritis—the presence of underlying occult neoplastic infiltration within the abdominal wall tissue must be considered a compounding local factor.

When evaluating long-term pathogenesis, several mechanisms must be distinguished. While iatrogenic tract implantation of tumor cells during her open laparotomy in 2002 remains the primary hypothesis, a delayed hematogenous abdominal wall metastasis cannot be definitively ruled out. Alternatively, this lesion could represent an isolated peritoneal implant that slowly invaded the adjacent fascia and posterior abdominal wall over decades, or a completely new primary peritoneal LGSC arising independently from the specialized extraovarian mesothelium of the hernia sac.

This pathological finding also underscores the need for patient counseling. Patients should understand the malignant potential of the initial pathologic diagnosis, the possibility of future transformative disease, and the rationale for long-term follow-up beyond the usual 5-year cancer surveillance. Ultimately, this case highlights the possibility of extremely late recurrence and supports individualized long-term follow-up in selected patients.

For general surgeons, this case shows the need for vigilance when operating on patients with a history of gynecologic surgery. If the contents of the hernia sac are unusual, signs such as scar thickening or unexplained symptoms should prompt submission of tissue for histopathologic evaluation. Surgeons must obtain a detailed clinical history and ensure prompt multidisciplinary referral to gynecologic oncology if a Müllerian malignancy is identified [3–7].
